# *PEG1/MEST* and *IGF2* DNA methylation in CIN and in cervical cancer

**DOI:** 10.1007/s12094-013-1067-4

**Published:** 2013-06-18

**Authors:** A. C. Vidal, N. M. Henry, S. K. Murphy, O. Oneko, M. Nye, J. A. Bartlett, F. Overcash, Z. Huang, F. Wang, P. Mlay, J. Obure, J. Smith, B. Vasquez, B. Swai, B. Hernandez, C. Hoyo

**Affiliations:** 1 Department of Obstetrics and Gynecology, Program of Cancer Detection, Prevention and Control, Duke University School of Medicine, P.O. Box 104006, Durham, NC 27710 USA; 2 Division of Gynecologic Oncology, Department of Obstetrics and Gynecology, Duke University School of Medicine, Durham, NC 27708 USA; 3 Department of Obstetrics and Gynecology, Kilimanjaro Christian Medical Centre, Tumaini University, Moshi, Tanzania; 4 Division of Infectious Diseases, Department of Medicine and Duke Global Health Institute, Duke University School of Medicine, Durham, NC 27710 USA; 5 Duke Comprehensive Cancer Center, Duke University Medical Center, Durham, NC 27710 USA; 6 Department of Epidemiology, University of North Carolina at Chapel Hill, Chapel Hill, NC 27599 USA; 7 Kilimanjaro Christian Medical Centre, Tumaini University, Moshi, Tanzania; 8 Tanzania and Duke Women’s Health Collaboration, Durham, NC 27710 USA; 9 Department of Pathology, Kilimanjaro Christian Medical Centre, Tumaini University, Moshi, Tanzania; 10 Cancer Research Center of Hawaii, University of Hawaii, Honolulu, HI 96822 USA

**Keywords:** Insulin-like growth factor 2 gene (*IGF2*), Paternally expressed gene 1/mesoderm-specific transcript (*PEG1/MEST*), Human papillomavirus (HPV), Cervical intraepithelial neoplasia (CIN), Invasive cervical cancer (ICC), Differentially methylated regions (DMRs)

## Abstract

**Introduction:**

Although most invasive cervical cancer (ICC) harbor <20 human papillomavirus (HPV) genotypes, use of HPV screening to predict ICC from HPV has low specificity, resulting in multiple and costly follow-up visits and overtreatment. We examined DNA methylation at regulatory regions of imprinted genes in relation to ICC and its precursor lesions to determine if methylation profiles are associated with progression of HPV-positive lesions to ICC.

**Materials and methods:**

We enrolled 148 controls, 38 CIN and 48 ICC cases at Kilimanjaro Christian Medical Centre from 2008 to 2009. HPV was genotyped by linear array and HIV-1 serostatus was tested by two rapid HIV tests. DNA methylation was measured by bisulfite pyrosequencing at regions regulating eight imprinted domains. Logistic regression models were used to estimate odd ratios.

**Results:**

After adjusting for age, HPV infection, parity, hormonal contraceptive use, and HIV-1 serostatus, a 10 % decrease in methylation levels at an intragenic region of *IGF2* was associated with higher risk of ICC (OR 2.00, 95 % CI 1.14–3.44) and cervical intraepithelial neoplasia (CIN) (OR 1.51, 95 % CI 1.00–2.50). Methylation levels at the *H19* DMR and *PEG1/MEST* were also associated with ICC risk (OR 1.51, 95 % CI 0.90–2.53, and OR 1.44, 95 % CI 0.90–2.35, respectively). Restricting analyses to women >30 years further strengthened these associations.

**Conclusions:**

While the small sample size limits inference, these findings show that altered DNA methylation at imprinted domains including *IGF2/H19* and *PEG1/MEST* may mediate the association between HPV and ICC risk.

## Introduction

Invasive cervical cancer (ICC) is the second most common cancer in women worldwide, with the heaviest burden borne by women in developing countries [1]. In the USA, more than 12,000 new ICC cases and 4,290 ICC-related deaths were reported in 2010 [2]. Incidence was 60 % higher and mortality was more than two times higher in Hispanics and African Americans compared with whites [1], despite comparable screening prevalence across racial/ethnic subgroups and comparable prevalence of infection with high-risk human papillomavirus (HR-HPV) [3]. Current screening guidelines recommend liquid-based cytology screening methods followed by testing for HR-HPV genotypes, since most ICCs evolve slowly and harbor HR-HPV genotypes [4]. However, the sensitivity and specificity of existing screening tests remains suboptimal, leading to false positives and overtreatment which have been linked to subfertility and cervical stenosis [5]. Molecular features that together with known environmental factors can improve prediction of progression of cervical intraepithelial neoplasia (CIN) to ICC, are required.

DNA methylation has been extensively investigated in human cancers and other disorders with the aim of improving present diagnostic tools [6], but appropriate target sequences are unknown. Predictions based on genome-wide approaches have been proposed as reviewed by Wentzensen et al. [7]; however, findings have been difficult to replicate or interpret, because DNA methylation patterns are cell-type specific and may be transient [7]. DNA sequences at differentially methylated regions (DMRs) that regulate imprinted genes (a group of genes that are defined by expression from only a single parental allele) offer key advantages. DNA methylation profiles that regulate these genes are normally stably maintained throughout somatic cell division [8]. In cancer, imprinted DMRs are often deregulated, showing gains or losses in methylation that affect changes in expression and sometimes imprint status [9]. DNA methylation changes can result in either silencing of the only active allele or, alternatively, aberrant activation of the normally silenced allele, which may double the gene dosage [10]. Given that about 80 % of known imprinted genes are found in clusters [8], altered methylation at a DMR can have wide-reaching effects within a cluster of genes.

The most intensively studied genomically imprinted gene, *Insulin*-*like Growth Factor 2* (*IGF2*), encodes for IGF2, a potent mitogenic growth factor, the expression of which is controlled by DMRs located in the *IGF2/H19* locus at chromosome 11p15.5. *IGF2* is maternally imprinted and expressed from the paternal allele, while the *H19* gene is maternally expressed. At the *IGF2/H19* locus, aberrant methylation at DMRs that regulate the expression of *IGF2* have been associated with *IGF2* deregulation in many cancers, including colorectal, pancreatic and ovarian cancers and Wilms’ tumors [11, 13]. We have also identified a novel consensus binding site for the zinc finger protein, CTCF, located in *IGF2* intron 3, and confirmed binding of CTCF to this region. The consensus sequence contains a single CpG dinucleotide whose methylation is strongly positively correlated with expression levels of *IGF2* in ovarian cancer (Huang and Murphy, in press). In addition, *Paternally Expressed Gene 1/Mesoderm*-*Specific Transcript* (*PEG1/MEST*) is an imprinted gene located at chromosome 7q32.2 and is expressed from the paternal allele. *PEG1/MEST* encodes for an enzyme belonging to the α/β-hydrolase fold family [14]. The gene promoter of the active paternal allele of *PEG1/MEST* is hypomethylated while the transcriptionally silent maternal allele is hypermethylated, regulating the monoalleleic expression of *PEG1/MEST*. Deregulated methylation and/or expression of *PEG1/MEST* have been associated with invasive breast cancer [15] and uterine leiomyoma [16] in humans, and growth retardation, defective nurturing and increased lethality in mice [17].

We examine whether aberrant DNA methylation of the *PEG1/MEST* and *IGF2/H19* regulatory regions and of three other imprinted gene DMRs belonging to this imprinted gene cluster: *Pleomorphic Adenoma Gene*-*Like 1* (*PLAGL1*), *Neuronatin* (*NNAT*), *Delta*-*like homolog 1* and *noncoding Maternally Expressed Gene 3* (*DLK1/MEG3*), is associated with CIN and ICC, taking into account HPV infection.

## Materials and methods

### Study participants

Patient identification and enrollment procedures have been previously described [18, 19]. Briefly, between November 2008 and March 2009, eligible study participants (*n* = 249) were identified from the appointment books of the Reproductive Health Clinic (RHC) at KCMC, and the final dataset (86 % of eligible participants or *n* = 234) were women for whom questionnaires, CIN status, and HPV genotype data were available. The study was a collaboration between Duke University and the Reproductive Health Center at Kilimanjaro Christian Medical Centre (KCMC) in northern Tanzania, a cervical cancer prevention clinic funded by the World Health Organization (http://www.afro.who.int/en/tanzania/who-country-office-tanzania.html).

Study procedures were approved by Research Ethics Boards at KCMC and Duke University.

### Data collection

#### Questionnaires

A trained nurse-interviewer obtained informed consent from all participants and administered a 40-min in-person standardized questionnaire focusing on demographic information and gynecologic history.

#### Specimens

Two cervical scrapes were obtained from each participant using an Ayres spatula and Cytobrush and rinsed into Preserv-CytTM media (Hologic, Inc., Malborough, MA), as described [19].

#### Ascertainment of CIN and carcinoma

Pap smears and biopsy specimens were processed and read by the KCMC staff pathologist, using standard conventions and ASCCP guidelines as appropriate (http://www.asccp.org/ConsensusGuidelines/tabid/7436/Default.aspx). Cases were classified using the Bethesda classification system [20]. These clinical results were compiled and transferred securely to Duke University.

#### HPV genotyping

Purified DNA was obtained from ThinPrep^®^ specimens and homogenized aliquotted biopsies and shipped to the University of Hawaii Cancer Center. PGMY09/PGMY11 primers [21] were used in PCR to target a 450-bp region of the HPV L1 gene, as described [19].

#### Ascertainment of HIV-1 infection status

Peripheral blood samples were centrifuged to separate the plasma and buffy coat. Plasma samples were used to test for HIV-1 infection using two rapid methods (Capillus HIV-1/HIV-2, Trinity Biotech PLC, Bray, County Wicklow, Ireland, and Determine HIV-1/2, Abbott Laboratories, Abbott Park, IL). Reactive specimens were then tested using Western blot according to standard clinical practice (Genetic Systems HIV-1 Western blot kit, Bio-Rad, Hercules, CA) [22].

#### DNA methylation analyses

Genomic DNA was prepared from cells isolated from the cervical scrapes or from biopsy specimens using PureGene protocol reagents (Qiagen; Valencia, CA). Genomic DNA was then modified by treatment with sodium bisulfite using the Zymo Easy-96 DNA Methylation Kit (Zymo Research, Irvine, CA). Bisulfite treatment of denatured DNA converts unmethylated cytosines to uracils but leaves methylated cytosines unchanged. When amplicons are sequenced, cytosines from methylated CpG sites appear as cytosine, while unmethylated cytosines appear as thymine. Pyrosequencing was performed using a Qiagen Pyromark Q96 MD Pyrosequencing instrument (Qiagen, Valencia, CA). The primer sequences and pyrosequencing assay conditions for the *PEG1/MEST* DMR, *PLAGL1* DMR, *MEG3* DMR, *NNAT* DMR, *IGF2* DMR, and *H19* DMR and assay validation using defined mixtures of methylated and unmethylated bisulfite-modified genomic DNA are described in detail elsewhere [23]. Methylation data were 98 % complete.

The intragenic CTCF site was identified based on the 5′-CCGCNNGGNGNC-3′ consensus previously defined [24] and is located in intron 3 of NCBI RefSeq NM_001127598. Binding of CTCF to this region was confirmed in ovarian cancer cells using chromatin immunoprecipitation as described (Huang and Murphy, in press). For quantitative methylation analysis of the intragenic region of *IGF2*, we amplified bisulfite-modified DNAs using forward primer 5′-TTG GGA AGT TTT TGT TTG TTA GT-3′ and biotin-labeled reverse primer 5′-ACC CTC AAA CCA AAC CCT AA-3′ in a 25-μl reaction mixture containing 40 ng of bisulfite-modified template DNA, 1.5 mM MgCl_2_ and 0.12 μM of each primer using the PyroMark PCR Kit (Qiagen, Valencia, CA). The sequence analyzed includes two CpG dinucleotides: one located within the consensus CTCF site and a second CpG outside the consensus motif (5′…gacc **CG** cagggtggctg**CG**tcc…3′; consensus underlined). We validated assay performance for linearity and the ability to detect increasing amounts of methylation across the full range of the assay (0–100 % methylation) using defined mixtures of unmethylated and methylated bisulfite-modified genomic DNA (Epitect Bisulfite Controls, Qiagen). The methylation measured by pyrosequencing was highly correlated with the amount of methylated template included in these reactions (*R* ^2^ = 0.99; *p* < 0.0001).

#### IGF2 expression analysis

Total RNA was extracted from 21 flash frozen ICC biopsy specimens using RNA Stat-60 (AMS Biotechnology, Abingdon, UK). cDNA was synthesized from 2 μg total RNA by oligo-dT priming using SuperScript II Reverse Transcriptase (Invitrogen, Carlsbad, CA), as described [19].

### Statistical analyses

Demographic and gynecological variables included age, history of pregnancy, number of pregnancies with complications (collected as a continuous variable and later dichotomized), and history of hormonal contraceptive use (dichotomized based on responses to ever having used either oral contraceptives, an intrauterine contraceptive device, medroxyprogesterone acetate, or a levonorgestrel intrauterine system). Chi-square tests were used to compare controls to CIN and ICC.

We interrogated between 3 and 8 CpG sites per DMR: 3 for *IGF2*, 4 for *H19*, 4 for *MEST*, 6 for *PLAGL1*, 4 for *MEG3*-*IG*, 3 for *NNAT*, and 8 for *MEG3*. To determine if a single mean was representative of methylation fractions at the multiple CG dinucleotides within the region analyzed, principal components analysis (PCA) was used. Cronbach’s alphas for these regions were 0.95–0.99, suggesting that methylation of the CGs was sufficiently correlated, allowing a single mean to be used.

Women were classified as having HR-HPV if they tested positive for at least one HR-HPV genotype, as defined by current FDA-approved HPV molecular tests. High-risk genotypes include 16, 18, 31, 33, 35, 39, 45, 51, 52, 56, 58, 59, 66, and 68; LR genotypes are 6, 11, 26, 40, 42, 53, 55, 61, 62, 69, 70, 72, 73, 81, 82, 83, and 84. Multinomial logistic regression models were then used to evaluate the association between CIN/ICC and changes in DMR methylation, and adjusting for age, HPV infection, history of pregnancy, HIV-1 status, and hormonal contraceptive use. The association of potential confounders with CIN and cancer status was also evaluated using Chi-square tests. All statistical analyses were conducted in SAS 9.1 (SAS Institute, Cary, NC).

## Results

Table 1 summarizes the characteristics of the 234 study participants. In all, 148 women (63 %) were classified as controls (women with neither CIN nor ICC). The remaining 86 women (37 %) were classified as cases; of these, 48 women (21 %) had ICC, 17 (7 %) had either CIN2 or CIN3, and 21 (9 %) had CIN1. The median age was 45 years; women with no cervical abnormalities and those with CIN1 were younger [mean 40.3 years (SD = 9.9) and 35.7 years (SD = 12.2), respectively] than women with cervical carcinoma or high-grade CIN [mean 55.2 years (SD = 12.3) and 44.7 (SD = 9.8) years, respectively]. All women with ICC and CIN2/3 were parous versus 86 % of women with CIN1 and 95 % of controls. Hormonal contraceptive use was more frequently reported by CIN cases (59–76 %) and controls (68 %) than ICC cases (40 %). Almost all (89 %) women with cancer were positive for at least one HPV genotype whereas only a small proportion of controls (14 %) had detectable HPV.

**Table 1:** Description of the Tanzanian women study participants as characterized by average age, HPV status, pregnancy and hormonal contraceptive use histories

Characteristics	Control (*n* = 148) *n* (%)	CIN1 (*n* = 21) *n* (%)	CIN2/3 (*n* = 17) *n* (%)	Cancer (*n* = 48) *n* (%)	*p* value
Age (mean, SD)	40.3 (9.9)	35.7 (12.2)	44.7 (9.8)	55.2 (12.3)	<0.0001
History of pregnancy					
Yes	135 (94.8)	18 (85.7)	17 (100)	48 (100)	0.043*
No	12 (9.2)	3 (14.3)	0 (0.0)	0 (0.0)	
Missing	1 (3)				
Hormonal contraceptive use					
Ever	98 (67.6)	16 (76.2)	10 (58.8)	19 (39.6)	0.0029
Never	47 (32.4)	5 (23.8)	7 (41.2)	29 (60.4)	
Missing	3				
Any HPV					
At least 1 HPV	20 (14.1)	12 (66.7)	14 (87.5)	33 (89.2)	<0.0001
No HPV	122 (85.9)	6 (33.3)	2 (12.5)	4 (10.8)	
Missing	6	3	1	11	
HIV-1 Infection					
Seropositive	30 (20.3)	12 (57.1)	12 (70.6)	7 (14.6)	<0.0001
Seronegative	118 (79.7)	9 (42.9)	5 (29.4)	41 (85.4)	

* Using Fisher’s exact test

Table 2 summarizes odds ratios (ORs) and 95 % confidence intervals for the associations between *IGF2*, *IGF2* intron 3, *H19*, *PEG1/MEST*, *MEG3*, *PLAGL1* and *NNAT* methylation and CIN or ICC. When compared with controls, a 10 % decrease in methylation at the *IGF2* intron 3 region (methylation mean = 71.97, SD = 8.5) was associated with a two-fold increase in ICC risk (OR 2.00, 95 % CI 1.14–3.44), after adjusting for age, gravidity, HIV-1 status, HPV infection, and oral contraceptive use. The association between lower methylation at the *IGF2* intron 3 region and ICC risk remained essentially unchanged (OR 1.88, 95 % CI 1.10–3.33) when restricting analysis to women aged 30+ years. A similar pattern of association, albeit weaker, was also observed between a 10 % methylation decrease for the *IGF2* intron 3 region and CIN (OR 1.51, 95 % CI 1.0–2.50).

**Table 2:** Adjusted ORs and 95 % CI for the association between mean methylation of six imprinted gene DMRs and CIN (1–3) and ICC

	ORs (95 % CI)	
	CIN (1–3) *n* = 26	Cancer *n* = 33
*IGF2/H19* imprinted domain		
*IGF2* intron 3 CTCF3 DMR methylation^a^	1.51 (1.00–2.5)	2.00 (1.14–3.44)
*IGF*-*2* DMR methylation^a^	1.11 (0.70–1.76)	1.00 (0.63–1.61)
*H*-*19* DMR methylation^a^	0.99 (0.57–1.71)	1.51 (0.90–2.53)
*PEG1/MEST* DMR methylation^a^	1.21 (0.80–1.84)	1.44 (0.90–2.35)
*PLAGL1* DMR methylation^a^	1.06 (0.68–1.64)	1.01 (0.65–1.56)
*MEG3* DMR methylation^a^	0.88 (0.64–1.21)	1.02 (0.70–1.48)
*MEG3_IG* DMR methylation^a^	0.73 (0.42–1.27)	0.76 (0.46–1.26)
*NNAT* DMR methylation^a^	1.05 (0.74–.147)	0.94 (0.63–1.39)
HPV	24.17 (6.61–88.46)	48.96 (9.17–261.50)

Adjusted for age, ever being pregnant, HIV-1 status, HPV infection, and oral contraceptive use

^a^10 % unit DNA methylation. All women with cancer had a history of pregnancy

Higher DNA methylation levels at the *PEG1/MEST* DMR were also associated with ICC (OR 1.44, 95 % CI 0.90–2.35), and restricting analysis to women 30+ years revealed a somewhat stronger association (OR 2.13, 95 % CI 1.10–4.16). Associations between DNA methylation at the *IGF2* or *H19* DMRs and risk of ICC or its precursor lesions were in a similar direction albeit non-significant. Restricting the analyses to women older than 30 years did not alter these findings. No associations between DNA methylation and ICC, or CIN, were found at *PLAGL1*, *NNAT* and *MEG3* DMRs (Table 2). The direction and magnitude of these associations remained unchanged when we excluded CIN1 lesions.

To examine whether DNA methylation at the *IGF2* intron 3 region, and at the *IGF2*, *H19* and *PEG1/MEST* DMRs may mediate the association between HPV infection and ICC, we ran mixed models—to allow for unconstrained model entry of individual CpGs at each DMR—to evaluate associations between DNA methylation levels at each of these four DMRs and HPV infection, in which no HPV infection served as the baseline category (Table 3). We found that both HR-HPV and LR-HPV infections were statistically significantly associated with decreased DNA methylation at the *IGF2* intron 3 region (*β* = −8.7, SE = 1.75, *p* < 0.0001; *β* = −17.74, SE = 4.70, *p* = 0.0002, respectively) and at the *IGF2* DMR (*β* = −3.38, SE = 0.85, *p* < 0.0001; *β* = −4.88, SE = 2.17, *p* = 0.02, respectively). HR-HPV infection was associated with higher methylation at the *PEG1/MEST* DMR (*β* = 1.75, SE = 0.78, *p* = 0.02). No associations were found for the *H19* DMR.

**Table 3:** Regression coefficients and standard errors (SE) for the association between HR-HPV and LR-HPV genotypes and DMR methylation

Chromosomal region and CpG site	High-risk HPV β-coefficient, SE, *p* value	Low-risk HPV β-coefficient, SE, *p* value
*IGF2* intron 3 CTCF3	−8.55, 1.75, <0.0001	−17.74, 4.70, 0.0002
*IGF2*	−3.38, 0.85, <0.0001	−4.88, 2.17, 0.02
*H19*	−0.04, 0.51, 0.93	−2.16, 1.27, 0.10
*PEG1/MEST*	1.75, 0.78, 0.02	2.80, 1.87, 0.13

Adjusted for multiple CpGs

No HPV infection served as the baseline category

To determine the functional significance of the association between DMR methylation at regulatory sequences of *IGF2*, we computed correlation coefficients for DNA methylation and log-*IGF2* gene expression from cervical cancer biopsies. We found higher IGF2 expression in samples where aberrant DNA methylation at *IGF2* regulatory regions was observed, compared to normal *IGF2* methylation at these same regions (*r* = 0.6, *p* = 0.001) (data not shown).

## Discussion

We examined the association between DNA methylation at sequences regulating the *PEG1/MEST*, *IGF2/H19*, *PLAGL1*, *MEG3* and *NNAT* imprinted domains and the risk of ICC and its precancerous lesions. After adjusting for age, lifetime gravidity, HIV-1 status, HPV infection, and oral contraceptive use, we found significant associations between altered DNA methylation levels at the *PEG1/MEST* and *IGF2/H19* imprinted domains, and risk of CIN and ICC. Our findings are consistent with the hypothesis that DNA methylation at regulatory sequences of imprinted genes represents susceptibility loci for ICC and its precursor lesions [7]. If confirmed in a larger number of these regulatory regions, these findings also suggest that DNA methylation, at these and perhaps other loci, may be developed for inclusion in risk stratification to identify individuals likely to progress from CIN to ICC.

The age at diagnosis in our Tanzania-based study is comparable to that of women in the USA and other developed countries, where ICC diagnosis tends to be in the fourth or fifth decades of life (SEER Stat Fact Sheets: Cervix Uteri), although this differs by racial/ethnic subgroup. In the USA, the age of peak incidence is 35–44 years in whites but 65+ in African Americans and Hispanics, despite minimal differences in Pap smear screening rates (whites 91.4 %, African Americans 89.7 %) [25]. Age has been shown to influence methylation marks and other regions such as gene promoters but not in DMRs regulating imprinted genes [26], regions previously thought to be resistant to the effects of aging [27]. Our finding of a stronger association in women aged 30+ are consistent with the findings of others [28] and suggests that DNA methylation marks in regulatory sequences of imprinted genes such as *PEG1/MEST* and *IGF2/H19* can be harnessed to improve prediction of CIN cases likely to progress in older women.

Although imprinted gene dysregulation has been shown to contribute to the development and progression of many human cancers [29], DNA methylation in DMRs regulating imprinted genes causally associated with altered expression of multiple genes within imprint gene clusters remains understudied. The most intensively studied imprinted gene, *IGF2*, has not yet been examined in relation to HPV infection and CIN progression. In the present study, we measured IGF2 transcript levels in women with ICC and found elevated IGF2 levels in samples where decreased methylation at the *IGF2* regulatory region was observed. Other studies have reported elevated IGF2 levels among women with cervical cancer and high- but not low-grade squamous intraepithelial lesions when compared with women with no history of abnormal Pap tests. Bulky tumors (>3 cm) are more common in women with higher IGF2 protein levels than in those with smaller tumors (<3 cm) [30]. It is possible that aberrant methylation in some CpGs in the *IGF2/H19* imprinted domain such as the intron 3 CTCF site may be influencing loss of imprinting and the exclusive usage of *IGF2* promoter 1, inducing *IGF2* overexpression, as has been previously shown in cervical carcinomas [30]. Albeit *IGF2* DMR aberrant methylation has been linked with other cancers [12, 13], this is the first time that *IGF2* intron 3 methylation has been examined in relation to cervical cancer.

Our findings of a strong association between HR-HPV infection and methylation levels support the hypothesis that aberrant DNA methylation may mediate the association between HPV infection and risk of CIN and ICC. If aberrant methylation occurs early in development, it would increase susceptibility to HPV infection and subsequently lesion progression, via elevated expression of these growth effectors—the *IGF2* and *PEG1/MEST* genes.

The study’s main limitation was its small sample size, which required combining CIN1-3 cases even though a sizable proportion of lower-grade CIN cases may not progress. A second limitation is that only a small fraction (<4 %) of Tanzanian women over 30 years of age are nulliparous (National Bureau of Statistics [Tanzania], ICF Macro. 2011), making parity-related sub-analyses difficult. Third, a larger sample size would enable testing the hypothesis of age-dependent DNA methylation in ICC. Finally, *PEG1/MEST* transcripts were not compared between normal and aberrant methylation as samples were limited; thus, the functional significance of aberrant DNA methylation can be inferred only from previous molecular data. However, the present data suggest that decreased methylation at the *IGF2* regulatory region upregulates IGF2 expression in cervical cancer tissue. Our study provides early empirical evidence for associations between DNA methylation patterns in regulatory sequences of imprinted gene and cervical cancer and its precursors. Despite these limitations, we found moderately strong associations between aberrant DNA methylation and ICC and its precursors, independent of known risk factors.

In summary, altered methylation at the *PEG1/MEST* DMR and at the *IGF2* intron 3 CTCF site was associated with increased ICC risk and HR-HPV infection. Larger studies are required to confirm our findings.
